# Invertebrate Decline Leads to Shifts in Plant Species Abundance and Phenology

**DOI:** 10.3389/fpls.2020.542125

**Published:** 2020-09-17

**Authors:** Josephine Ulrich, Solveig Franziska Bucher, Nico Eisenhauer, Anja Schmidt, Manfred Türke, Alban Gebler, Kathryn Barry, Markus Lange, Christine Römermann

**Affiliations:** ^1^ Institute of Ecology and Evolution, Friedrich Schiller University, Jena, Germany; ^2^ German Centre for Integrative Biodiversity Research (iDiv) Halle-Jena-Leipzig, Leipzig, Germany; ^3^ Institute of Biology, Leipzig University, Leipzig, Germany; ^4^ Department of Biogeochemical Processes, Max Planck Institute for Biogeochemistry, Jena, Germany

**Keywords:** flowering phenology, global change, iDiv Ecotron, insect decline, biotic interaction, global change experiment, peak flowering, trophic cascading

## Abstract

Climate and land-use change lead to decreasing invertebrate biomass and alter invertebrate communities. These biotic changes may affect plant species abundance and phenology. Using 24 controlled experimental units in the iDiv Ecotron, we assessed the effects of invertebrate decline on an artificial grassland community formed by 12 herbaceous plant species. More specifically, we used Malaise traps and sweep nets to collect invertebrates from a local tall oatgrass meadow and included them in our Ecotron units at two different invertebrate densities: 100% (no invertebrate decline) and 25% (invertebrate decline of 75%). Another eight EcoUnits received no fauna and served as a control. Plant species abundance and flowering phenology was observed weekly over a period of 18 weeks. Our results showed that invertebrate densities affected the abundance and phenology of plant species. We observed a distinct species abundance shift with respect to the invertebrate treatment. Notably, this shift included a reduction in the abundance of the dominant plant species, *Trifolium pratense*, when invertebrates were present. Additionally, we found that the species shifted their flowering phenology as a response to the different invertebrate treatments, e.g. with decreasing invertebrate biomass *Lotus corniculatus* showed a later peak flowering time. We demonstrated that in addition to already well-studied abiotic drivers, biotic components may also drive phenological changes in plant communities. This study clearly suggests that invertebrate decline may contribute to already observed mismatches between plants and animals, with potential negative consequences for ecosystem services like food provision and pollination success. This deterioration of ecosystem function could enhance the loss of insects and plant biodiversity.

## Introduction

Global warming and land-use changes alter ecosystems worldwide (Estes et al., 2011; Rasmann et al., 2014; Giling et al., 2019). Insect species go extinct (Dirzo et al., 2014; Sánchez-Bayo and Wyckhuys, 2019; Seibold et al., 2019), and the insect biomass decreases dramatically (Hallmann et al., 2017; Seibold et al., 2019). As a consequence, invertebrate community composition changes, as some invertebrates shift their distributions, causing alterations in co-occurrence patterns (Rasmann et al., 2014). Some future scenarios predict an increase in herbivory and herbivore pest outbreaks because of reduced top-down regulation due to missing key predators resulting from rising temperatures, nitrogen deposition, and habitat loss (Coley, 1998; Voigt et al., 2007; de Sassi and Tylianakis, 2012; Sánchez-Bayo and Wyckhuys, 2019; Seibold et al., 2019). Further, higher trophic levels may be more affected by environmental change than lower trophic levels (Coley, 1998; Voigt et al., 2007; de Sassi and Tylianakis, 2012). Thus, herbivorous invertebrates may benefit from both, a warmer climate favoring their developmental times and lower predator pressure, which may subsequently favor pest outbreaks (Coley, 1998).

In addition to altering insect community dynamics, climate and land-use changes also shift plant species abundances and flowering phenology. Alpine grasslands, for example, alter their plant community structure showing an increase in grass abundance due to rising temperatures (Liu et al., 2018). Grassland species from warm and temperate regions are also susceptible to anthropogenic land-use changes, such as fertilization, grazing and clipping (Borer et al., 2014; Hautier et al., 2014; Shi et al., 2015). In terms of their phenology, changes in first and last flowering day, flowering duration or peak flowering are all associated with an increase in temperatures (Menzel et al., 2006; Bock et al., 2014; Bucher et al., 2018; König et al., 2018; Bucher and Römermann, 2020). CaraDonna et al. (2014) documented temperature-driven shifts in plant communities over 39 years and stated that species-specific changes in phenology can alter temporal co-occurrence patterns. Previous findings revealed that some plant species advance or prolong their flowering period in response to changing climatic conditions or land use changes, whereas other species do not respond at all (Bock et al., 2014; CaraDonna et al., 2014; Moore and Lauenroth, 2017; Bucher et al., 2018; Bucher and Römermann, 2020).

However, climate change and land use do not only lead to phenological changes in plants, they also affect invertebrate phenology (Rathcke and Lacey, 1985; Root et al., 2003; Bartomeus et al., 2011; Burkle et al., 2013; Ovaskainen et al., 2013) and biotic interactions. In responses to rising temperatures, some bee species exhibited a larger shift in phenology than plants (Burkle et al., 2013), whereas certain solitary spring bees did not advance their phenology as much as their host plants (Kehrberger and Holzschuh, 2019). Biotic changes themselves, such as the loss of plant diversity (Wolf et al., 2017) and invertebrate biomass (Hallmann et al., 2017; Seibold et al., 2019), affect ecological relationships, e.g. plant-pollinator or competitive interactions which are related to plant fitness (Rathcke and Lacey, 1985; Visser and Both, 2005; Parmesan, 2007; Kehrberger and Holzschuh, 2019). As those plant-pollinator interaction networks seem to be less resilient to future changes, mismatches in biotic interactions are likely (Burkle et al., 2013).

Notably, multitrophic interactions, such as the relationship between plants and invertebrates, affect plant species abundance and phenology: For example, herbivore pressure is positively correlated with the number of flowers produced by a plant individual (Strauss et al., 1996). Poveda et al. (2003) reported shorter flowering durations as a response to increased herbivory and Trunschke and Stöcklin (2017) found an extension of flowering duration when pollinators were excluded. These findings provide empirical evidence for biotic interactions altering plant phenology. Consequently, these alterations may not only lead to mismatches in plant-insect interactions due to species loss and shifts in phenology but may also cause losses of ecosystem functions such as flower availability. However, despite this evidence for biotic interactions changing plant species abundance and phenology (Strauss et al., 1996; Poveda et al., 2003; Trunschke and Stöcklin, 2017; Kehrberger and Holzschuh, 2019), there have been few studies exploring potential invertebrate density effects on plant abundance and phenology.

This study aims at addressing this gap and identifying the link between invertebrate decline and plant species abundance and phenology. More specifically, we established 12-species grassland communities in 24 controlled Ecotron chambers (Eisenhauer and Türke, 2018) and with three different treatments simulating a decrease in invertebrate density by 0%, 75% and 100%. We used this experiment to answer the following questions: 1) Does a decrease in invertebrate density affect plant species composition? 2) Does a decrease in invertebrate density affect flower phenology? This research leads to a better understanding of the effects of changing invertebrate density on plant species composition and phenology in the future and evaluates the indirect effects that changes in land use may have on biodiversity.

## Materials and Methods

### Experimental Setup

The experiment was carried out at the iDiv Ecotron (Eisenhauer and Türke, 2018) at the research station of the Helmholtz-Centre for Environmental Research (UFZ) in Bad Lauchstädt, Germany (51° 22’ 60N, 11° 50’ 60E, 118 m a.s.l.). It is located in the Central German dry area (Querfurter Platte) with a mean annual temperature of 8.9°C (1896-2013) as well as a mean annual precipitation of 489 mm (1896-2013) (Schädler et al., 2019; Siebert et al., 2019). Here, we used 24 identical experimental units (EcoUnits) with controlled environmental conditions such as light, air, and soil temperature, and irrigation (Eisenhauer and Türke, 2018). The EcoUnits further allowed us to observe the vegetation *via* two HD-IP-video cameras per EcoUnit which provided pictures taken at two different angles. Taken together, they captured at a minimum 50% of each EcoUnit. The cameras took one picture every day at 18:00 CEST with a resolution of 2688*1520 (4085760 pixels). Outdoor seasonal changes regarding the day length and temperature were mimicked. One EcoUnit combined 1.2 m^3^ of standardized soil mixture (see below) and a usable air space of about 2 m^3^. The soil surface was a square of 1.5 m^2^. The outer dimensions of one EcoUnit was 1.55 m × 1.55 m × 3.20 m (L × W × H). For the belowground part, the internal dimensions was 1.24 m × 1.24 m × 0.80 m (L × W × H) and for the aboveground part it was 1.46 m × 1.46 m × 1.50 m (L × W × H). Prior to the experiment, the EcoUnits were filled with sieved (15 mm mesh size) top soil (80%) and sand (20%) mixture purchased by commercial suppliers (LAV Technische Dienste GmbH & Co.KG, Erdwerk Kulkwitz). Approximately 20 kg of soil from the sites where invertebrate sampling was carried out (see below) was added to each EcoUnit to inoculate soil organisms, such as soil microorganisms, microfauna (e.g. nematodes), and mesofauna (e.g. Collembola and mites), to establish a similar soil invertebrate community in the EcoUnits. This grassland site was formerly used as an arable field, where the last crop cultivation happened in 2012. The soil of the Querfurter Platte is a Haplic Chernozem, which has a high fertility and was developed on carbonatic loess substrate containing 70% silt and 20% clay. Values for pH ranged from 5.8 to 7.5, the total carbon content varied between 1.71% and 2.09% and total nitrogen ranged from 0.15% to 0.18%, in the upper 15 cm (Schädler et al., 2019; Siebert et al., 2019). Abiotic conditions of all EcoUnits were optimized to provide suitable growth conditions for the target plants: day time ranged from 5:00 to 21:00 with transitions from 0% illumination at 4:00 to 100% at 6:00 and 100% illumination at 20:00 to 0% at 22:00. The average air temperature at 30 cm above the ground level during the daytime was 24°C and changed to 19°C on average at night. Due to extreme hot weather which overheated the building the maximum temperature reached 28°C in the afternoon for a period of 10 days from May 20^th^. The average soil temperature at 9 cm below soil surface was 18°C. The irrigation volume was 6 l of de-ionized water per day per EcoUnit including an overflow at the edges. The same amount of viable seeds for each of 12 selected plant species (**Supplementary Table 1**, three grasses, nine herbs) belonging to a tall oatgrass meadow (Arrhenatherion elatioris) was directly sown into EcoUnits, equalling a total of 1,320 seeds (n = 1,000 viable seeds per m^2^ of plant growth area). We chose species that are insect pollinated (except for the grasses which are predominantly pollinated by wind) and which are known to flower in the first year after sowing based on experience from a biodiversity experiment, the so called Jena Experiment (Weisser et al., 2017) located in 70 km distance to the Ecotron. The seed material was provided by Rieger Hofmann GmbH, Blaufelden-Raboldshausen, Germany, and was chosen from origin area No. 2 “Mitteldeutsches Tief- und Hügelland” after the rules of the Association of German Wild Seeds Producers. The species-specific numbers of viable seeds were calculated based on thousand grain weight and adjusted to germination rates, which were assessed in the laboratory beforehand. Therefore, 30 seeds of a single species were sown in a tray filled with the same soil that we used for the experiment with two replicates per species (n = 60 seeds). The seeds were not scarified prior to seeding. Germlings were counted and removed daily for a period of 22 days. Using germination rates, the required amount of seeds referring to an equal number of 110 viable seeds per species were mixed and applied regularly in EcoUnits. The seeds were sown on April 19^th^ 2018. The experiment ended on November 15^th^ 2018. This analysis includes data from April 26^th^ to August 20^th^ 2018, as there was a mid-term harvest after August 20^th^ 2018. Thus, we had a study period of 18 weeks.

To control for potential effects of soil nutrients on plant abundance and phenology, the plant-available nutrients were examined in the soil solution. Soil solution was sampled using suction cups with a diameter of 20 mm, a length of 50 mm, a bubble point 0.89 bar, and an average pore size of 1 µm (Umwelt-Geräte-Technik GmbH, Müncheberg, Germany) four times during the study period of 18 weeks. The sampling bottles were continuously evacuated to a negative pressure of -20 kPa. Cumulative soil water was sampled fortnightly and processed immediately for measuring the concentrations of dissolved inorganic nitrogen species (NO_3_^-^ and NH_4_^+^), phosphate (PO_4_^3-^), and potassium (K^+^). Measurements for NO_3_^-^ and PO_4_^3-^ were performed on an ion chromatography system DX-500 (Thermo Fisher Scientific GmbH, Dreieich, Germany), while NH_4_^+^ and K^+^ were quantified on an ion chromatography system ICS-5000 (Thermo Fisher Scientific GmbH, Dreieich, Germany). The soil nutrient analyses revealed no difference across the treatments and over time (**Supplementary Table 2**).

**Figure 1:**
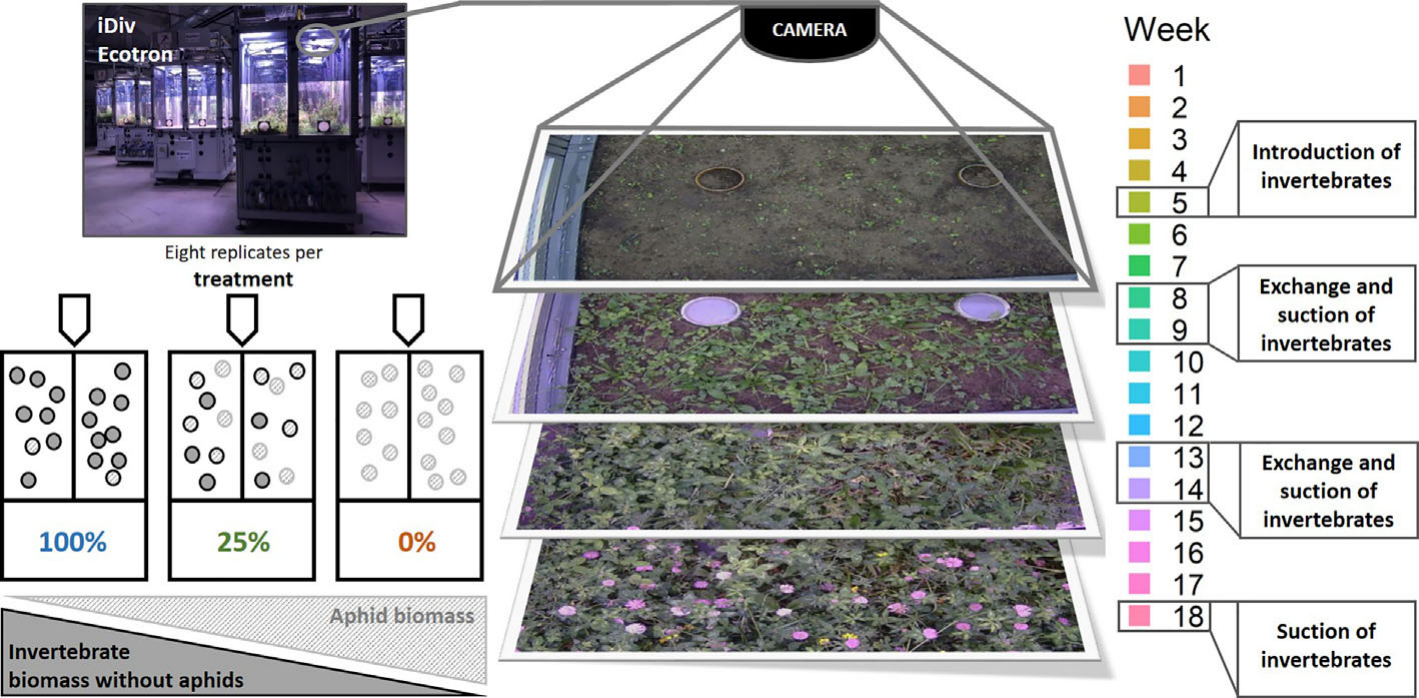
Set up of the Ecotron experiment to assess the effect of a loss of invertebrate biomass on plant species composition and phenology of an experimental 12-species grassland community. The photo in the top-left corner shows the iDiv Ecotron (Eisenhauer and Türke, 2018). In each EcoUnit, cameras were installed which took daily pictures of the vegetation (middle). Different treatments were applied to simulate changes in invertebrate biomass (100%, 25%, and 0%) with eight replicates each (bottom left). A decrease in invertebrate biomass negatively correlated with aphid biomass (bottom left and **Supplementary Figure 1**). As indicated in the timeline (right), invertebrate introduction took place after 5 weeks, invertebrate exchange after 8 and 13 weeks. The last suction marked the end of our experiment.

To assess the effect of declining invertebrate densities on plant species abundance and phenology, three different invertebrate treatments (100%, 25%, 0%) were established with eight replicate EcoUnits each. Invertebrates were caught on adjacent oatgrass meadows of the research station in Bad Lauchstädt using Malaise traps (tall end height: 1.7 m, short end height: 0.9 m, width: 1.15 m, length: 1.88 m) with a catching height of 1 m to capture all plant visiting invertebrates, and sweep nets to catch the invertebrates directly from the vegetation. For both catching methods, we applied different catching efficiencies corresponding to two different invertebrate densities: 100% and 25%. The 100%-treatment simulated a situation without any invertebrate decline with respect to current local conditions, while the 25%-treatment corresponded to a 75% decline of the current local conditions. In addition, we added a 0%-treatment, in which no invertebrates were added. To assess the true area-specific biomass of invertebrates at the sampling site, we took suction samples using cages of the dimensions of the aboveground part of an EcoUnit (1.5 m * 1.5 m). The invertebrates, which were caught with Malaise traps and sweep nets, were introduced into the EcoUnits of the corresponding treatments 5 weeks after seed sowing when plant leaves were fully developed. To simulate natural species turnover, invertebrates were removed and replaced with newly collected specimens after eight and 13 weeks, respectively (**Figure 1**). To remove the invertebrates from the units, a modified commercial vacuum cleaner (Bosch Industriestaubsauger GAS 25) was used following a standardized procedure that defined a specific time frame of equal length for the extraction from one segment (four per EcoUnit). In parallel, invertebrates were caught in adjacent meadows as described above and introduced during the next period (**Figure 1**). Notably, this suction of invertebrates was applied to all EcoUnits to keep the disturbance levels constant across the treatments. After 18 weeks of the experiment, a third invertebrate sampling was applied in all EcoUnits. Thus, we had three sampling dates, where the invertebrates were identified and weighed. As we used the biomass to analyze the invertebrate treatment (see below), we will refer to it by using invertebrate biomass instead of invertebrate density.

Even though the establishment of the invertebrate treatments was successful, we observed aphid infestations (Aphidina species), which increased in severity from the 100%- to the 0%-treatment and from week 5 to week 18 (**Figure 1**, **Supplementary Figure 1**). As aphids appeared to be a substantial driver of the treatment and to support the interpretation of our results, we assessed patterns in aphid biomass and diversity between the treatments. Analyses and results are provided in the **Appendixes S1** and **S2**. Aphid biomass was significantly different across the treatments after the first sampling event (**Supplementary Figure 1**). Total invertebrate biomass and invertebrate biomass excluding aphids showed the highest values after the last sampling. We found that aphids represented a high proportion of the total invertebrate biomass in the 25%- and 0%-treatment. A significant difference between treatments was detected for the proportion of aphids in the total biomass. Regarding the invertebrate diversity, the Shannon diversity revealed significant differences between the 100%- and 0%-treatment after the first sampling (**Supplementary Figure 2**, for statistical procedure with respect to the treatment see supplementary). Soil invertebrates were also present in the invertebrate sampling (cf. **Supplementary Figure 2**) but were not analyzed separately.

### Changes in Plant Species Abundance and Phenology

Based on standardized camera pictures, we estimated plant species abundance and phenology every week. Using the cameras was necessary, because we could not open the EcoUnits as invertebrates could have escaped or been transferred from one EcoUnit to another. We took the pictures every Thursday, as the picture series started on a Thursday, and only switched to Wednesdays when the pictures taken on a Thursday were blurred. As the mid-term harvest started the day after August 20^th^, the last day of data sampling was a Monday. For each picture of an EcoUnit, we performed vegetation relevés using the Schmidt-Scale (1974, cited in Pfadenhauer, 1997) for plant species abundance estimations with one additional class for very low abundances: 0, 0.5, 1, 3, 5, 8, 10, 15, 20, 25, 30, 40, 50, 60, 70, 75, 80, 90, and 100%. For each picture and species, we also estimated the percentage of flowers in a population using the same scale to capture the first flowering day and the peak flowering. That is, on the population level we estimated the proportion for the vegetative stage, the flower buds, the flowers and the end of flowering, so that taken together we described the phenological stages of the population for 100% for every week. Of the 12 plant species sown, we could only record seven species (**Table 1**). *Bellis perennis* L. and *Knautia arvensis* (L.) Coult. did not flower, grew only very occasionally underneath the plant cover, and were therefore not visible in the camera pictures. It was not possible to identify the grass species from the pictures even though at least some individuals flowered.

**Table 1:** Overview of observed plant species with corresponding abbreviation, family, life from, pollination syndrome, flowering time, and the information whether the species flowered during the experiment.

Species	Abbreviation	Family	Life form	Pollination syndrome	Flowering time	Flowered
*Centaurea jacea* L. s. l.	Cen_jac	Asteraceae	Hemicryptophyte	Insects	Jun–Nov	yes
*Lotus corniculatus* L.	Lot_cor	Fabaceae	Hemicryptophyte	Insects	Jun–Aug	yes
*Medicago lupulina* L.	Med_lup	Fabaceae	Hemicryptophyte	Insects, self-pollination	May–Oct	yes
*Plantago lanceolata* L.	Pla_lan	Plantaginaceae	Hemicryptophyte	Wind, insects, self-pollination	May–Oct	yes
*Scorzoneroides autumnalis* (L.) Moench	Sco_aut	Asteraceae	Hemicryptophyte	Insects	Jul–Sep	yes
*Trifolium pratense* L.	Tri_pra	Fabaceae	Hemicryptophyte	Insects	Jun–Sep	yes
*Achillea millefolium* L.	Ach_mil	Asteraceae	Hemicryptophyte	Insects	Jun–Oct	no

Information based on Klotz et al. (2002) and our personal observations.

### Statistical Analyses

#### Changes in Plant Species Abundance

To explore the general effects of week and the treatment on the vegetation, we conducted a principal component analysis (PCA) on the scaled and centered data of plant species abundance per treatment and week (as captured by the different pictures) using the “vegan” package (Oksanen et al., 2019) in R (R Core Team, 2018). Prior to the PCA, we checked that axis length was <3 performing a detrended correspondence analysis (DCA) following the procedure described in Leyer and Wesche (2007). With the “envfit”-function, we correlated the variables week and treatment with the PCA axes. To better visualize temporal changes in species composition per treatment, centroids were calculated as mean values grouped by week and treatment for the first and second principle components.

To analyze the effect of week and treatment on changes in species abundance, we used boosted regression trees (BRTs) using the R package “gbm” (Greenwell et al., 2019) and the modified functions provided by Elith et al. (2008). This is a machine learning approach based on regression trees, where data transformation or elimination of outliers is not needed (see Elith et al., 2008 for further details). We ran a model for each species separately and for every plant functional group, including treatment as a factor. We used the following parameter settings: A Gaussian error distribution, as we dealt with proportional data, a tree complexity of 2, a bagging fraction of 0.5, and a learning rate of 0.01. The models were fitted with the “gbm.step”-function and assessed with the cross-validation correlation (cv). The cv is also used to show withheld portions of the data (Elith et al., 2008).

#### Changes in Plant Species Phenology

We assessed general patterns in flowering phenology using multivariate statistics and conducted a DCA as the length of the gradient was >3, as described above. With the “envfit”-function, we correlated week and treatment (in %) with the axes. We calculated the centroids as the mean grouped by week and treatment for the first and second DCA-axis.

To analyses effects of invertebrate biomass on plant species phenology, we used BRTs similar to the procedure we followed for the abundance. For each species, the percentage of flowers was included as the response variable, week, and treatment were explanatory variables. We used the same parameter settings that were applied for the BRTs of the abundance analyses (see above).

For the graphical presentation of all plots, we used the package “ggplot2” (Wickham, 2016).

## Results

### Changes in Plant Species Abundance

**Figure 2:**
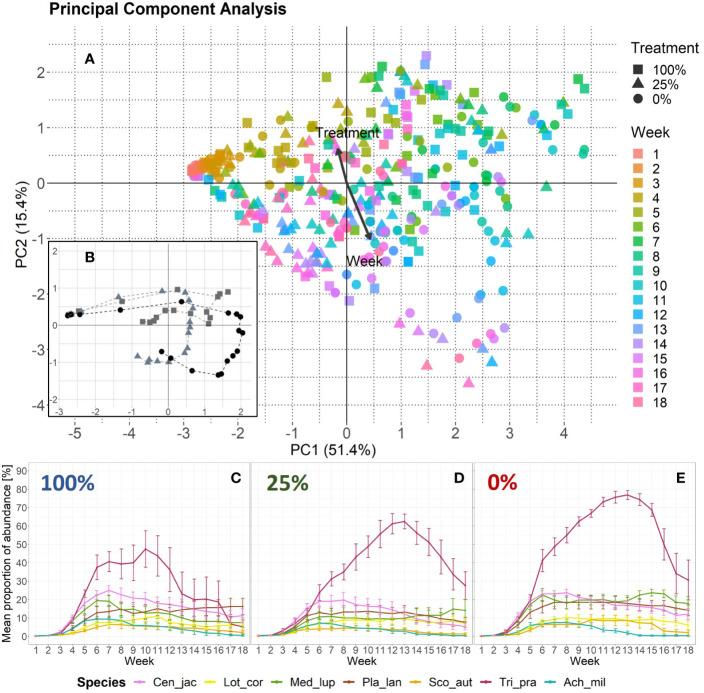
Changes in plant species abundance over time per treatment. **(A)** Principal component analysis based on species abundance data. One data point represents one EcoUnit of the iDiv Ecotron observed in 1 week (n = 431). The community development over time (18 weeks) is indicated by a color gradient representing the week. Eigenvalues are given in percent and represent the explained variance according to the axes. The treatments are represented by filled symbols (square = 100%, triangle = 25%, circle = 0%). Variables week and treatment are post-hoc correlated (p < 0.001). Arrows are enlarged in scale by the factor two to fit the scale of the plot. Their lengths show differences in explained variance relative to each other. **(B)** PCA-centroids per week and treatment. The dashed lines connect the symbols that represent the plant community abundance development over time. **(C**, **D)** Plant species-specific changes in abundance over time as given by mean proportion of percentage values with standard error. Each invertebrate treatment is represented by a panel: **(C)** 100%-treatment, **(D)** 25%-treatment, and **(E)** 0%-treatment. The seven plant species are color-coded. Species abbreviations are listed in **Table 1**.

The PCA revealed that temporal changes in plant species composition differed between treatments and that week appeared to be more important than treatment as indicated by the longer vector in **Figure 2A**. However, the analysis also showed a separation along the PC2-axis (**Figure 2B**) which was correlated with the invertebrate treatment gradient. When we compared changes in species abundances across weeks and treatments, we found that according to the relative importance values given by the species-wise BRT models, week explained from 66.7% in *Medicago lupulina* to 94.8% in *Lotus corniculatus* (**Supplementary Figure 3A**). However, treatment explained from 5.2% in *L. corniculatus* to 33.3% in *M. lupulina*. The values for the cross-validation correlation ranged from 0.49 for *Scorzoneroides autumnalis* to 0.78 for *T. pratense*. When we included aphid biomass from the three sampling dates (**Supplementary Figure 1A**) as an additional independent variable, the cross-validation correlation was higher, that is from 0.74 for *Achillea millefolium* to 0.91 for *T. pratense*, and the relative importance of aphid biomass (ranging from 34.4% in *A. millefolium* to 56.6% in *S. autumnalis*) was similar to the relative importance of week (ranging from 32.8% in *S. autumnalis* to 54.8% in *A. millefolium*) across all plant species (**Supplementary Figure 3B**). However, treatment was still important. The most abundant species in all treatments was *T. pratense*, yet its abundance increased from the 100%- to the 0%-treatment (**Figures 2C–E**). Furthermore, this species decreased earlier in the 100%-treatment (week 10) as compared with the other treatments (week 13).

**Figure 3:**
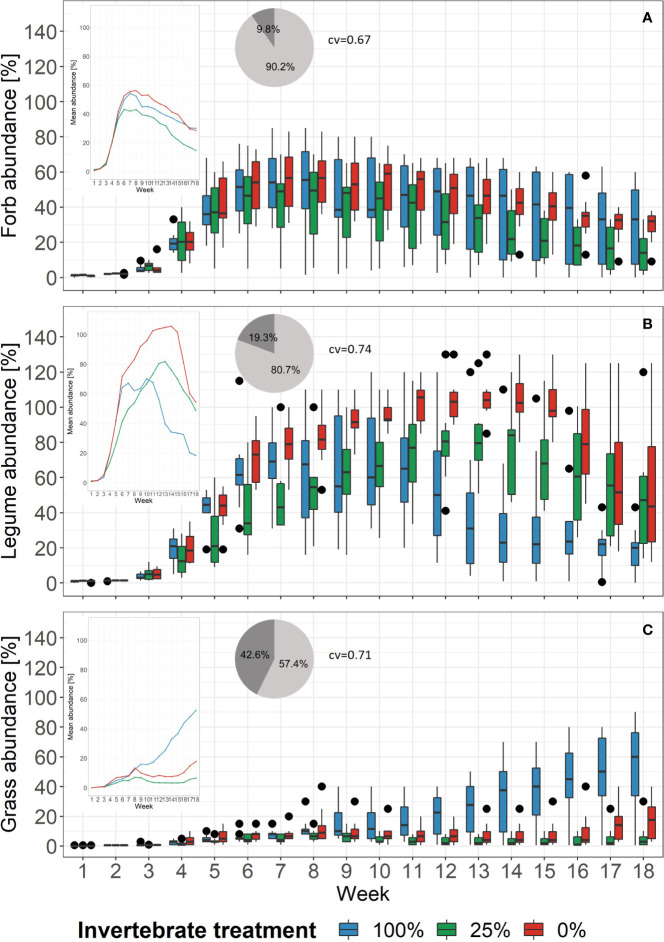
Changes of the proportion of abundance of plant functional groups in each treatment over time. Black dots mark outliers. Small windows give the mean proportion of abundance of the same functional group as lines. Pie charts show the relative importance based on boosted regression trees of the variables week (light grey) and treatment (dark grey). Right beside it, the cross-validation correlation of the models is given (cv). See **Supplementary Figure 8** for partial dependence plots. **(A)** Forb abundance, **(B)** Legume abundance, **(C)** Grass abundance.

The abundance of plant functional groups over time and per treatment is given in **Figure 3**. The cv that derived from BRTs ranged from 0.67 for forbs to 0.71 and 0.74 for grasses and legumes, respectively. The abundances of forbs over time showed similar patterns across the treatments. The BRT models revealed that the treatments’ relative importance was 9.8% (**Figure 3A**). Legume abundance, however, decreased earlier in the 100%-treatment as compared to the 25%- and 0%-treatment (**Figure 3B**). Here, the BRT showed that treatment had a relative importance of 19.3%. For the grasses the trend was *vice versa*: The grass abundance increased in the 100%-treatment and stayed relatively low in the 25%- and 0%-treatment (**Figure 3C**). The BRT revealed a relative importance of 42.6% for treatment. For the functional groups the cv increased as well when including aphid biomass as a third explanatory variable, with 0.73 for legumes, 0.86 for forbs, and 0.91 for grasses (**Supplementary Figure 4B**). The differences in relative importance between week and aphid biomass was similar for forbs and grasses. Forbs had a relative importance value of 46.4% for week and 44% for aphid biomass. Grasses showed 36.6% for week and 43% for aphid biomass. However, the relative importance of 1% for aphid biomass was the lowest compared with all BRTs.

### Changes in Plant Species Phenology

**Figure 4:**
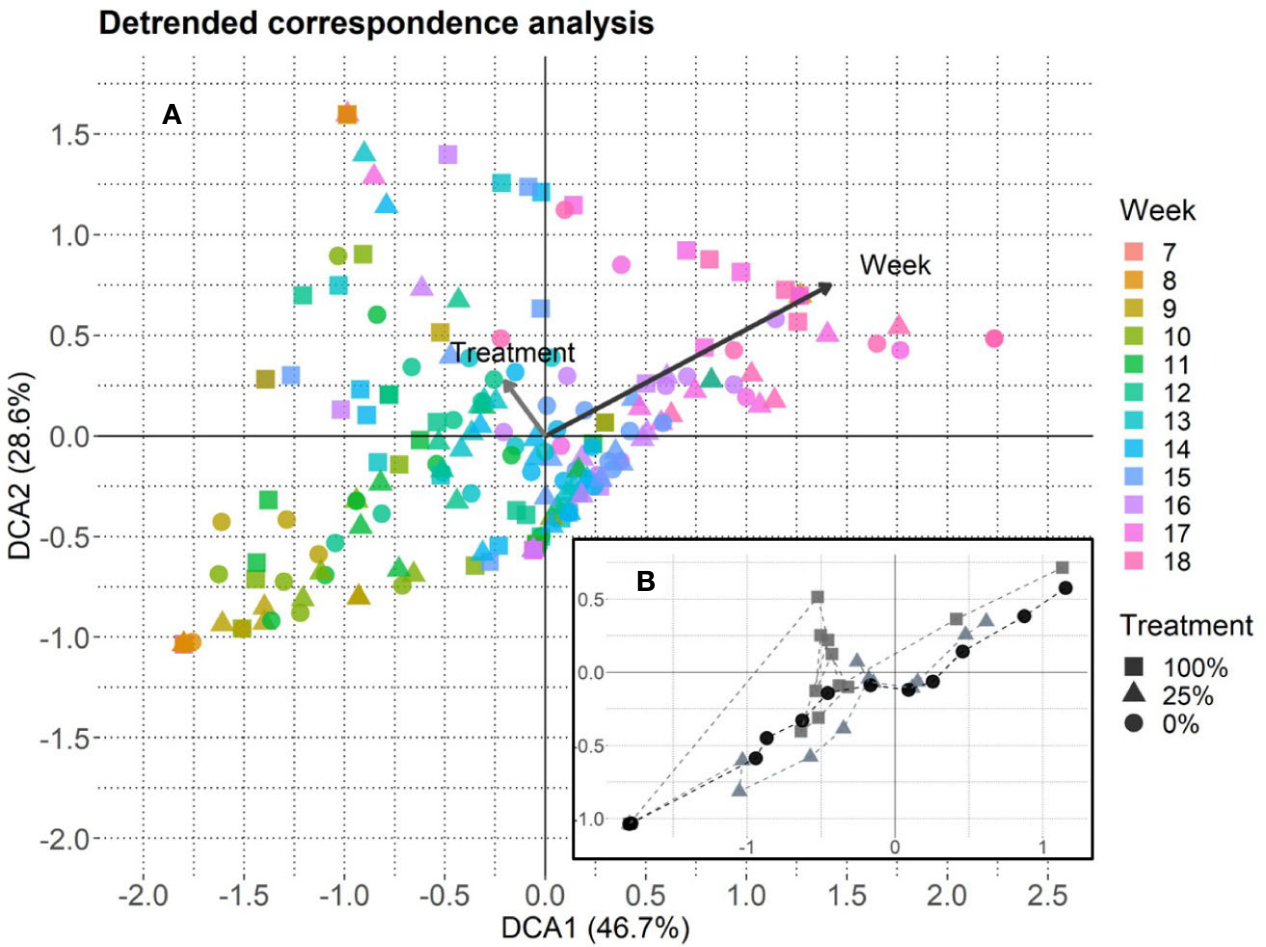
Detrended correspondence analysis based on species-specific flowering data, axis lengths: DCA1 **=** 4.03 and DCA2 **=** 2.63. **(A)** One data point represents one EcoUnit observed in 1 week (n = 228). The community development over time (18 weeks, starting from week 7 when plant species started to flower) is given with a color gradient for week. Eigenvalues are given in percent and represent the explained variance according to the axes. The treatments are represented by filled symbols (square = 100%, triangle = 25%, circle = 0%). Variables week and treatment are post-hoc correlated (p < 0.001). Arrows are enlarged in scale by the factor two to fit the scale of the plot. Their lengths show differences in explained variance relative to each other. **(B)** DCA-centroids per week and treatment. The dashed lines connect the symbols that represent the plant community flowering development over time.

**Figure 5:**
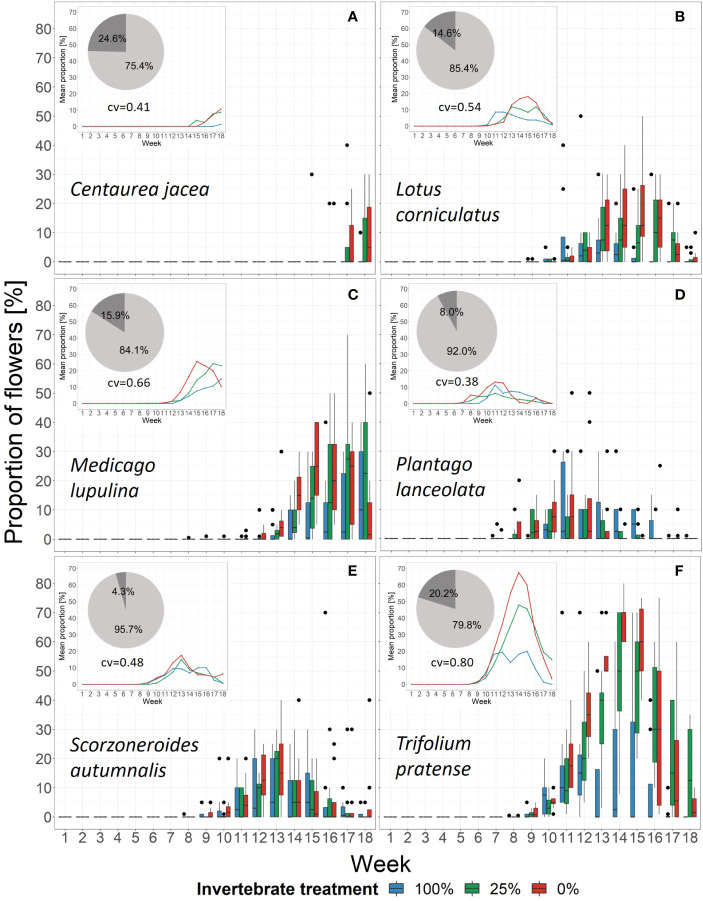
Changes in the proportion of flowers of the six plant species in each treatment over time. Black dots mark outliers. Small windows give the mean proportion of the same species within the community as lines. Pie charts show the relative importance based on boosted regression trees of the variables week (light grey) and treatment (dark grey). Underneath the pie chart the cross-validation of the models is given (cv). See **Supplementary Figure 7** for partial dependence plots. **(A)** *Centaurea jacea*, **(B)** *Lotus corniculatus*, **(C)** *Medicago lupulina*, **(D)** *Plantago lanceolata*, **(E)** *Scorzoneroides autumnalis*, **(F)** *Trifolium pratense*.

The DCA revealed that week mainly drove flowering phenology and that treatment had a marginal influence (**Figure 4A**). The DCA-centroids per week and treatment showed that the data points for the 100%-treatment developed along the treatment gradient (**Figure 4B**). On the community level, the peak flowering tended to show a higher dispersion in the 100%-treatment (week 11 to 18), that converged in the 25%- (week 11 to 17) and the 0%-treatment (week 11 to 15; cf. **Figure 5**). However, the coefficient of variation did not significantly differ between treatments, even though a higher variation was detected for the 100%-treatment compared to the 25%- and 0%-treatment (**Supplementary Figures 5A, B**).

The BRT models showed that the relative importance of week was higher than treatment in every species. In addition, treatment also influenced flowering phenology patterns over time, even though its importance differed among species. It was highest for *C. jacea* (24.6%) and lowest for *S. autumnalis* (4.3%). The cross-validation correlation showed that on the species-level between 41% in *C. jacea* and 80% in *T. pratense* of the variation was explained by variables week and treatment (**Figure 5** and **Supplementary Figure 3C**). When we included aphid biomass as an explanatory variable, the cross-validation correlation was higher (ranging between 0.63 in *S. autumnalis* and 0.86 in *T. pratense*), but nevertheless, treatment still had an influence (**Supplementary Figure 3D**). However, plant phenology responses to the treatment were species-specific (**Figure 5**). C. *jacea* tended to flower earlier in the 25%- and 0%-treatment than in the 100%-treatment. However, as there was only one flowering observation for this species in the 100%-treatment a significance test could not be applied (**Supplementary Figure 5D**). For *M. lupulina*, we found a delayed first flowering in the 100%-treatment compared to the 0%-treatment (**Supplementary Figure 5D**). This species tended to reach average peak flowering in week 15 in the 0%-treatment, after 17 weeks in the 25%-treatment while it did not reach peak flowering in the 100%-treatment (**Figure 5**). This relationship was opposite in *L. corniculatus*: on average, peak flowering took place earlier (in week 11) in the 100%-treatment and was delayed in the other treatments (**Figure 5**). Nevertheless, across all species peak flowering time only differed significantly across treatments for *L. corniculatus* which reached peak flowering earlier in the 100%-treatment compared to the 0%-treatment, and *P. lanceolata* which showed a later peak flowering for the 100%-treatment compared to the 25%-treatment (**Supplementary Figure 5C**). The species *S. autumnalis* did not respond to the treatment. There was also no difference in peak flowering time of *T. pratense* with respect to the treatment, however, we detected a higher proportion of flowers in the 25%- and 0%- than in the 100%-treatment (**Figure 5**).

## Discussion

The results of this experiment demonstrated that in addition to the widely studied abiotic drivers like climate variables (Menzel et al., 2006; Bock et al., 2014; Bucher et al., 2018; König et al., 2018), biotic components are able to drive the abundance and phenological changes of plant species. We found that a decline of invertebrate biomass led to species-specific changes in plant species abundances over time in experimental plant communities, and to species-specific responses in the proportion of flowers. The effect of the three invertebrate treatments was apparent. However, the changes detected may not only result from decreased invertebrate densities, but may also be attributed to the changed composition regarding the ratio of predators and aphids.

### Changes in Plant Species Abundance

Changes in plant species composition during the course of the experiment suggested that the plots became more similar to their initial state towards the end of the project (“circular movement”), which was mainly driven by proceeding experimental time, as plant species developed and disappeared due to their life cycles. However, the invertebrate treatment also influenced the plant species abundance, which was mainly driven by the dominance of *T. pratense* among all EcoUnits. This is also a dominant species in semi-natural mesophilic grasslands due to its ability to efficiently use limiting resources (Roscher et al., 2008). The abundances of this species decreased earlier in time for the 100%-treatment, and other species, such as *P. lanceolata*, became dominant during the last weeks of the experiment. These community changes could be a predator-mediated effect: invertebrate predators can have an indirect positive effect on plant species abundance, as their presence reduces herbivores and thus the feeding pressure on the plants (Messina, 1981; Schmitz et al., 1997; Carson and Root, 1999; Schmitz et al., 2004; Preisser and Bolnick, 2008). The shifts in plant functional groups, such as the suppression of grasses in the 25%- and 0%-treatment compared to the 100%-treatment, could have been mediated by the abundance of carnivorous invertebrates which controlled the number of herbivores or led to their behavioral changes (Werner and Peacor, 2003). Schmitz et al. (1997) demonstrated that under a low predation risk, generalist grasshoppers predominantly feed on nutritious grasses and shift their feeding to less nutritious herbs in response to rising predation risk. As grasshoppers occurred in low numbers and the grass cover constantly increased in the 100%-treatment whilst in the other treatments it remained at lower levels, we could assume that the plant consumption was more uniformly distributed across the plant species due to intact interaction networks in the 100%-treatment. Changes in the plant community composition may also be a response to changes in the soil invertebrate community. The high relative number of Collembola species, which we observed at the beginning of the experiment and which decreased over time but remained relatively high for the 100%-treatment (**Supplementary Figure 2**), indicated a change in this community may modify plant species abundance, as these decomposers are known to affect, e.g. plant growth (Partsch et al., 2006; Eisenhauer et al., 2011).

### Changes in Flowering Phenology

Regarding the flowering phenology, the EcoUnits did not diverge with respect to the invertebrate treatment as shown in the DCA, yet species-specific changes within each treatment revealed strong differences. *Trifolium pratense* was not only the most dominant species, but also showed highest percentages of flowers together with *L. corniculatus*. This intense flowering may reflect a response to stress caused by higher herbivore pressure. For other herbaceous plant species, Strauss et al. (1996); Strauss (1997), and Poveda et al. (2003) suggested that they tended to increase the number of flowers per individual as a reaction to leaf herbivory, and that herbivore-induced foliar damage tended to delay flowering. In our experiment, we assumed that there was higher herbivore pressure in EcoUnits with more aphids (and lower invertebrate biomass and species diversity). We found that peak flowering of the plant species community tended to be more condensed for the 25%- and 0%-treatment where the herbivore pressure was higher as compared with the 100%-treatment where peak flowering tended to be more dispersed. This trend was however, not statistically significant. Another possible explanation for the species-specific flowering patterns could be that certain plant species, e.g. *C. jacea* or *L. corniculatus*, are not self-compatible and depend on invertebrates as pollinators, whereas others do not. The herb *P. lanceolata* is zoochorous and anemochorous, as well as self-compatible (cf. **Table 1**) and may be more resilient in terms of its flowering pattern to changes in the invertebrate community as pollinators are not obligatory for its reproduction (Clifford, 1962; Friedman and Barrett, 2009). A similar relationship was hypothesized for the first flowering days in trees in a global meta-analyses (König et al., 2018) and for herbs along elevational gradients (Bucher and Römermann, 2020). For *S. autumnalis*, there was no clear pattern. This species is also insect pollinated, but as the curves of the 25%- and 0%-treatment were relatively similar, it could be possible that this perennial plant preferred to spare its resources for the next flowering period. Plant species which depend on insect pollination and are not pollinated are expected to extend their flowering period to enhance pollination success (Alonso, 2004; Castro et al., 2008; Aronne et al., 2015). We speculate that the best compromise for the plant species in this study was to invest in a higher number of flowers to increase pollination success, but not in flowering duration. This potential trade-off could be a subject of future studies. Compared to the herbivores, we only had a low amount of pollinators in the invertebrate communities. In the study by Veits et al. (2019), it was shown that plants reacted to insect sounds by an increase in nectar content and were thus able to sense their pollinators. If plants were aware of the presence of pollinators, the differing peak flowering times across the species in the 100%-treatment could be seen as a response to the occurrence of pollinators, while their absence in the 0%-treatment may have resulted in a convergence of community peak flowering. This phenological complementarity has positive effects in nature as it provides food for pollinators over a longer time period and reduces pollinator competition (Stiles, 1975; Lobo et al., 2003). A reduction of invertebrate densities may lead to a shorter community flowering period which may result in a mismatch of biotic interactions (Goulson et al., 2015; Schenk et al., 2018). As we did not analyze mismatches in more detail, further studies are needed to investigate the degree of changes in biotic mismatches with changing invertebrate densities.

### Effects of Abiotic Factors on Plant Species Abundance and Phenology

Previous studies showed that higher legume abundances and concomitant higher nitrogen rhizodeposition can result in differing soil nutrient conditions (Jensen, 1996; Fustec et al., 2010), eventually leading to species-specific responses: It has been shown that a reduction in the availability of nutrients promotes flowering in *Arabidopsis thaliana* (L.) Heynh. and *Pharbitis nil* (L.) Roth (Shinozaki et al., 1988; Kolář and Seňková, 2008; Wada et al., 2009; Wada and Takeno, 2010; Cho et al., 2017). In our experiment, the soil nutrient analyses did not significantly differ across treatment and over time and could thus not explain differences in the flowering pattern. Thus, the earlier peak flowering in *L. corniculatus* in the 100%-treatment could not be an effect of lower availability of nutrients. The response of *M. lupulina* to the invertebrate treatment was the reverse of the response of *L. corniculatus*. This species showed a very dispersed flowering pattern across the treatments with the peak flowering appearing first in the 0%-treatment, followed by the 25%- and the 100%-treatment. Turkington and Cavers (1979) reported a clear edaphic effect regarding the flowering of *M. lupulina* as this species delays its flowering with lower pH values. Maybe the pH changed throughout the experiment and this abiotic factor could explain the delay in first flowering of this species.

### Caveats of the Study and Implications for Future Studies

A combination of different effects such as herbivore pressure, higher amount of available nutrients or the presence of pollinators, resulted from the invertebrate treatments and could have been responsible for the observed patterns in plant species abundance and phenology. The treatment of a reduced invertebrate biomass resulted in a loss of predators and thus in an increase of aphid biomass in the 25%- but also and especially in the 0%-treatment, where invertebrates appeared even though they were not introduced. Hence, the reduction of invertebrate biomass in our treatment led to significant changes in the invertebrate community represented by a concomitant reduction of invertebrate diversity (**Supplementary Figures 1** and **2** ). Thus, we need to consider that the experimental treatments led not only to changes in the invertebrate biomass but also to a reduction of predator species, which worked as natural pest controls. This pattern, however, might actually reflect the consequences of the globally observed invertebrate decline: other studies have shown that an increase in temperature alters invertebrate communities in a way that herbivorous insects are favored as their developmental times decrease when the top-down regulations are reduced (Coley, 1998; de Sassi and Tylianakis, 2012; Rasmann et al., 2014) and the sensitivity of organisms to the effects of climate change increases with trophic rank (Voigt et al., 2007). Consequently, pest outbreaks are more likely in a warmer future (de Sassi and Tylianakis, 2012). Altered top-down forcing regimes associated with missing high trophic level consumers (Estes et al., 2011) might have caused the patterns in plant species abundance and phenology that we could observe in our experiment, because responses to changes in interaction networks happen relatively fast (Burkle et al., 2013). Thus, further research is needed to disentangle the effects of invertebrate densities and trophic structure on plant species communities. Expanding the study by considering also soil invertebrate communities would shed light on an underrepresented but highly influential field of interaction research (Wardle et al., 2004; Eisenhauer and Türke, 2018). In addition, soil nutrient conditions potentially explain shifts in flowering time. Another approach to explain the changes in abundance and phenology patterns may be the analysis of plant functional traits, e.g. if the shorter flowering period in the 0%-treatment leads to a resource allocation within the plant leaves as a trade-off effect. Simonsen and Stinchcombe (2014) could show that higher levels of insect damage increased leaf nitrogen for *M. lupulina*. This study gave a first impression on how a reduced invertebrate density could influence ecosystem functions with respect to plant species abundance in combination with flower availability. The research on plant functional traits could enlarge the knowledge about how plants adapt to declining invertebrate densities within and between species and how this may shape ecosystem functions.

## Conclusion

Our results showed that changes in invertebrate communities significantly affected the abundance and phenology of plant species in a species-specific way. We observed distinct shifts in species abundance and flowering phenology as a response to the different invertebrate treatments. The shifts in plant species abundances and phenology as a response to abiotic conditions such as rising temperatures may be promoted by changing biotic components like the already observed invertebrate decline. These changes may contribute to mismatches of interactions between invertebrates and plants. Consequences could be a reduced pollination that may result in both, a lack of energy provision for pollinators and a lower reproduction success in plants. A higher abundance of herbivores in response to reduced top-down control by predators leads to more damage on plant tissue, and pollinators were shown to visit damaged plants less frequently (Strauss et al., 1996; Strauss, 1997). Thus, the decline of invertebrates may lead to a further loss of plant species. Future research is required exploring the underlying mechanisms, such as changes in mutualistic and antagonistic interactions between invertebrates and plants to disentangle the specific drivers that caused the patterns in plant species abundance and phenology we observed in our experiment. The results of this study highlight the effects of an under-appreciated driver of plant abundance and phenology with considerable impacts on ecosystem functions, namely changes in invertebrate communities.

## Data Availability Statement

The datasets generated for this study are available on request to the corresponding author.

## Author Contributions

The Ecotron experiment was conceived, established, and maintained by NE, MT, AS, and AG. The idea of this study was developed by CR and SFB. The observations of plant species abundance and phenology was conducted by JU. The analyses were performed by JU. The text was written by JU. CR and SFB contributed significantly to statistical as well as graphical design ideas and to the text. AS provided the table for the overview of the invertebrates that were introduced to the EcoUnits and sucked out, as well as identified afterwards. ML provided soil nutrient data. All authors contributed to the article and approved the submitted version.

## Funding

Financial support came from the German Centre for Integrative Biodiversity Research Halle-Jena-Leipzig, funded by the German Research Foundation (FZT 118).

## Conflict of Interest

The authors declare that the research was conducted in the absence of any commercial or financial relationships that could be construed as a potential conflict of interest.
