# Supplementary material for: Invertebrate Decline Leads to Shifts in Plant Species Abundance and Phenology
Source: Front Plant Sci. 2020 Sep 17;11:542125. doi: 10.3389/fpls.2020.542125 (PMC7527414; doi:10.3389/fpls.2020.542125)
Supplement: Supplementary file 1 [file DataSheet_1.docx]

Assessment of invertebrate treatment

We assessed the differences in the applied invertebrate treatment by comparing the invertebrate biomass, the aphid biomass and the Shannon diversity. Here we tested for significant differences with respect to the sampling date, as we had three invertebrate suction events (Figure 1). In order to test for differences across the treatments for every sampling date, we performed an ANOVA followed by pairwise t-tests using Holm method when test conditions (normal distribution and homoscedasticity) were met. When normality or homoscedasticity were rejected, we performed a Kruskal-Wallis test with subsequent pairwise Wilcoxon tests. The same tests were conducted to compare peak flowering and first flowering across treatments and species.
