# Supplementary material for: Invertebrate Decline Leads to Shifts in Plant Species Abundance and Phenology
Source: Front Plant Sci. 2020 Sep 17;11:542125. doi: 10.3389/fpls.2020.542125 (PMC7527414; doi:10.3389/fpls.2020.542125)
Supplement: Supplementary file 2 [file DataSheet_2.docx]

Assessment of soil water nutrients

Linear mixed-effects models (LME) applying the ‘lme’-function in the R library ‘nlme’ (Pinheiro et al. 2016) were used to test for invertebrate effects on nutrient concentrations. Starting from a constant null model, with ‘EcoUnit’ as random intercept accounting for the repeated measurements at the same EcoUnits, the null model was extended stepwise. The fitting sequence started with ‘invertebrate treatment’ (factorial term), followed by ‘time’ of sampling, and the interactions between ‘invertebrate treatment’ and ‘time’. The maximum likelihood method was used and likelihood-ratio tests (L-Ratios) were applied to assess the statistical significance of stepwise model improvement.
