# Supplementary material for: Invertebrate Decline Leads to Shifts in Plant Species Abundance and Phenology
Source: Front Plant Sci. 2020 Sep 17;11:542125. doi: 10.3389/fpls.2020.542125 (PMC7527414; doi:10.3389/fpls.2020.542125)
Supplement: Supplementary file 3 [file Image_1.pdf]

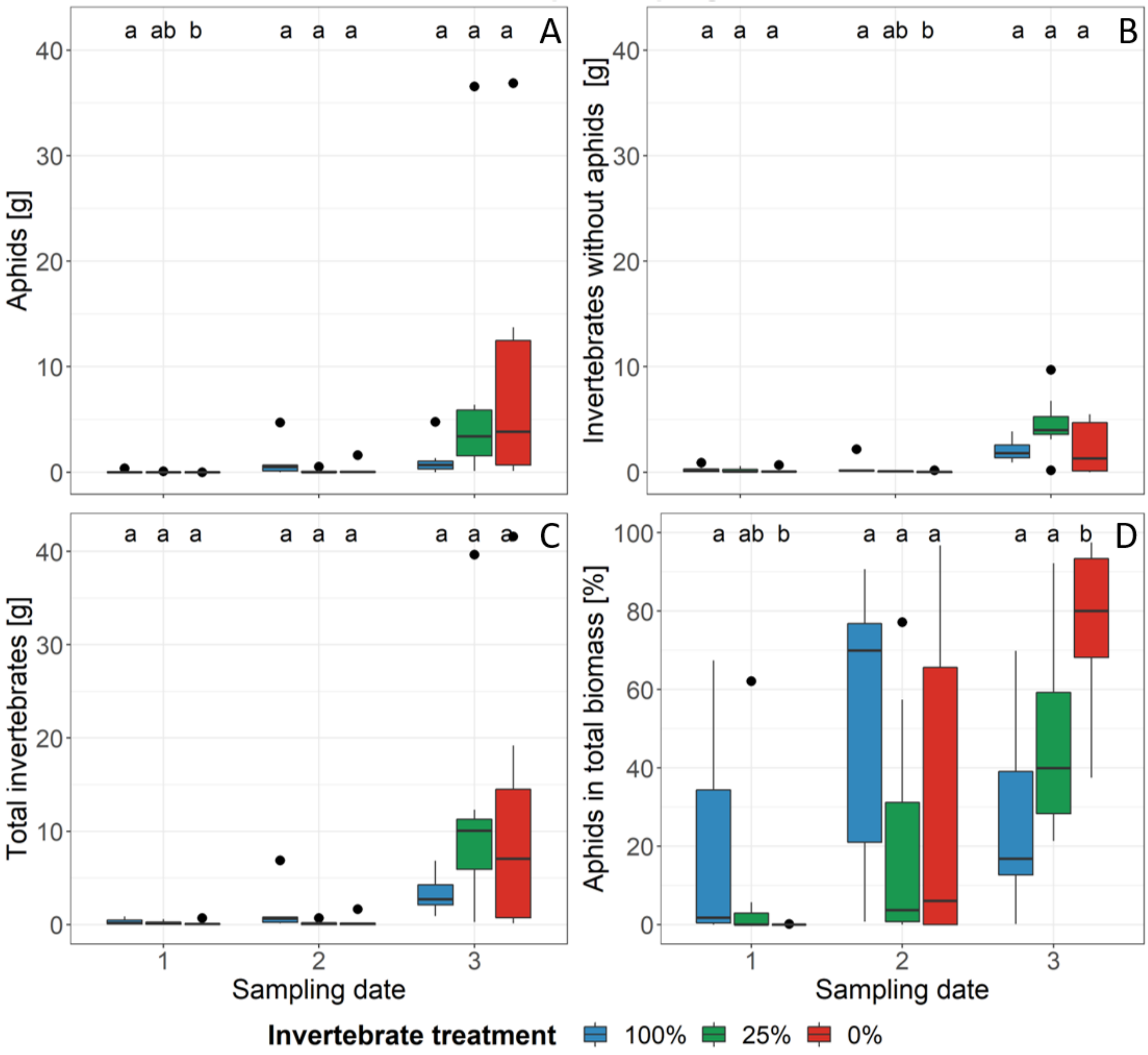

Supplementary Figure 1. Invertebrate biomass per sampling date. Significant differences were calculated for each sampling date separately. Invertebrate treatment is color-coded. (A) Aphid biomass, sampling date 1: Kruskal-Wallis test χ^2=7.33, df=2, p<0.05, sampling date 2: Kruskal-Wallis test χ^2=4.66, df=2, p=0.10, sampling date 3: Kruskal-Wallis test χ^2=4.14, df=2, p=0.13, (B) Invertebrates without aphids, sampling date 1: Kruskal-Wallis test χ^2=0.25, df=2, p=0.88, sampling date 2: Kruskal-Wallis test χ^2=7.44, df=2, p<0.05, sampling date 3: Kruskal-Wallis test χ^2=4.60, df=2, p=0.10, (C) Total invertebrates, sampling date 1: Kruskal-Wallis test χ^2=1.01, df=2, p=0.60, sampling date 2: Kruskal-Wallis test χ^2=5.84, df=2, p=0.05, sampling date 3: Kruskal-Wallis test χ^2=3.03, df=2, p=0.22. (D) Aphids in total biomass, sampling date 1: Kruskal-Wallis test χ^2=7.60,

df=2, $p<0.05$, sampling date 2: Kruskal-Wallis test $\chi^2=2.28$, df=2, $p=0.24$, sampling date 3: ANOVA $F=9.86$, df= 2, $p<0.001$.
