# Supplementary material for: Invertebrate Decline Leads to Shifts in Plant Species Abundance and Phenology
Source: Front Plant Sci. 2020 Sep 17;11:542125. doi: 10.3389/fpls.2020.542125 (PMC7527414; doi:10.3389/fpls.2020.542125)
Supplement: Supplementary file 4 [file Image_2.pdf]

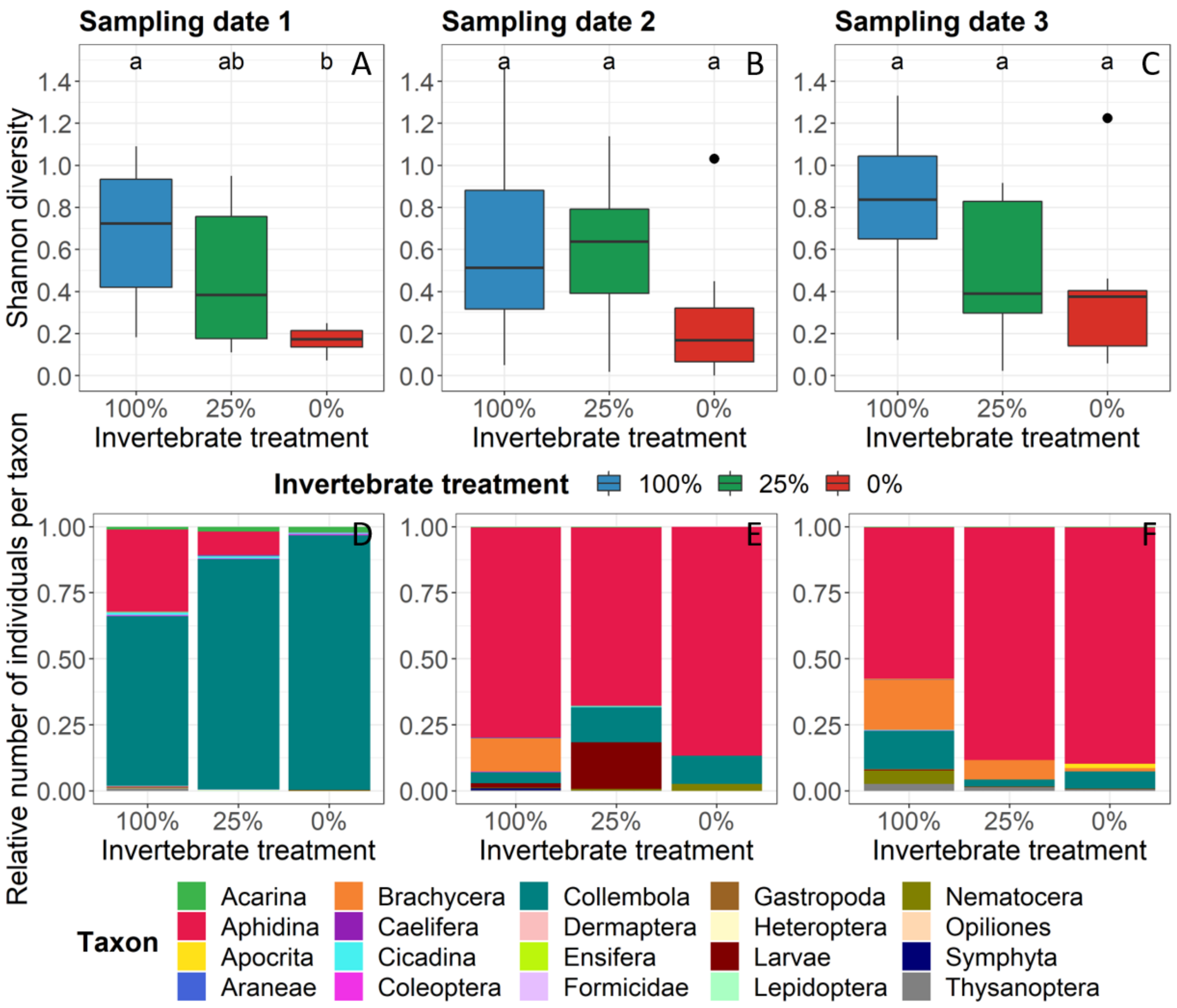

Supplementary Figure 2. Shannon diversity and relative number of individuals per taxon according to the three sampling dates. (A) Boxplots with Shannon diversity for sampling date 1, Kruskal-Wallis test χ^2=7.08, df=2, p<0.05, (B) Boxplots with Shannon diversity for sampling date 2, Kruskal-Wallis test χ^2=4.38, df=2, p=0.11, (C) Boxplots with Shannon diversity for sampling date 3, ANOVA F=2.99, df=2, p=0.07. (D-F) Bar chart showing the relative number of individuals per invertebrate taxon. The taxa are color-coded.
