# Supplementary material for: Invertebrate Decline Leads to Shifts in Plant Species Abundance and Phenology
Source: Front Plant Sci. 2020 Sep 17;11:542125. doi: 10.3389/fpls.2020.542125 (PMC7527414; doi:10.3389/fpls.2020.542125)
Supplement: Supplementary file 5 [file Image_3.pdf]

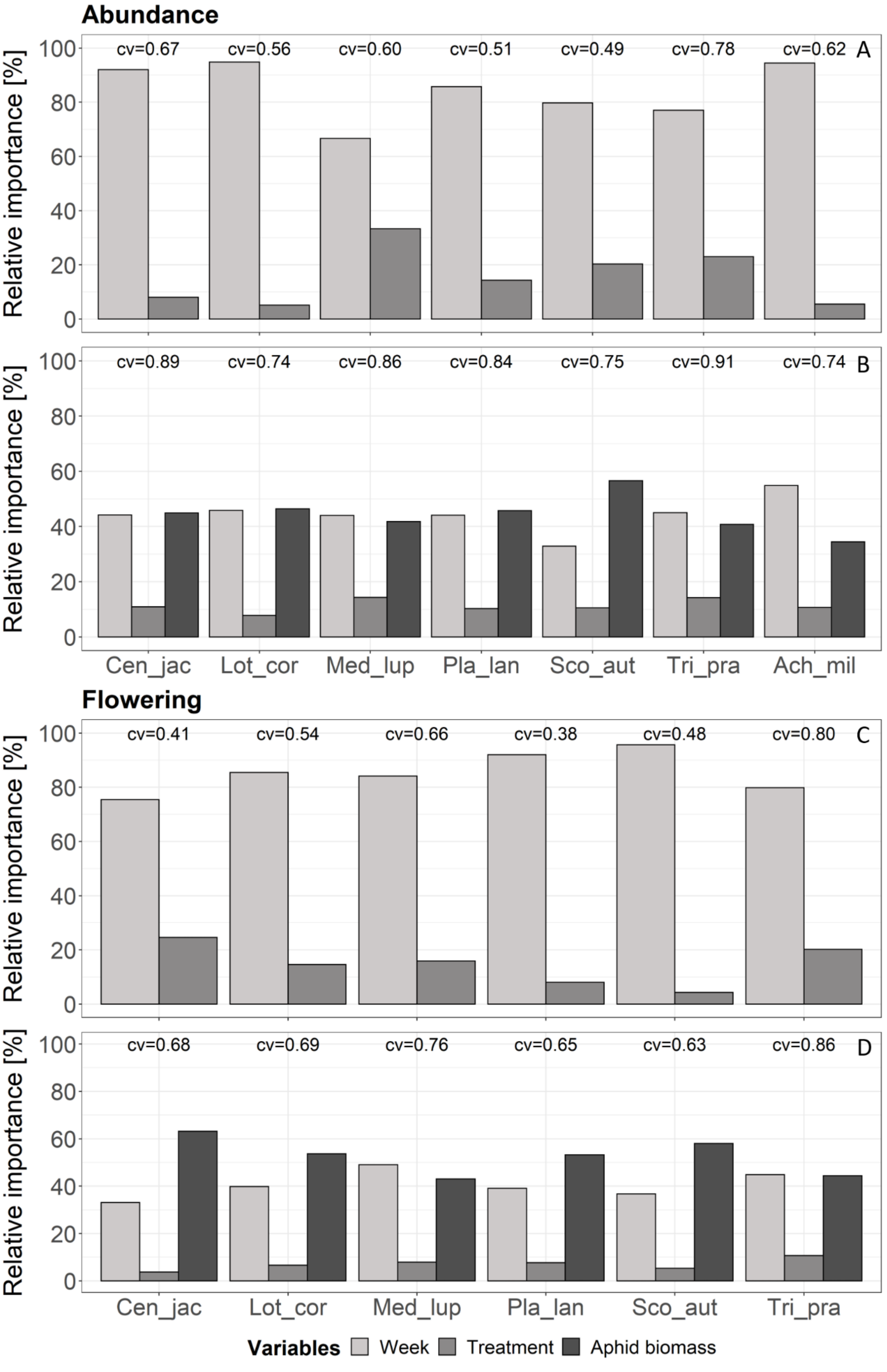
Abundance
Flowering
Relative importance [%]
cv=0.67
cv=0.56
cv=0.60
cv=0.51
cv=0.49
cv=0.78
cv=0.62
A
cv=0.89
cv=0.74
cv=0.86
cv=0.84
cv=0.75
cv=0.91
cv=0.74
B
Cen_jac
Lot_cor
Med_lup
Pla_lan
Sco_aut
Tri_pra
Ach_mil
cv=0.41
cv=0.54
cv=0.66
cv=0.38
cv=0.48
cv=0.80
C
cv=0.68
cv=0.69
cv=0.76
cv=0.65
cv=0.63
cv=0.86
D
Variables
Week
Treatment
Aphid biomass

Supplementary Figure 3. Bar charts representing the relative importance of the variables week, treatment and aphid biomass for each species, deduced from boosted regression tree (BRT) models, according to abundance (7 species with 432 observations) and flowering (6 species with 432 observations); see text for parameter settings and Table 1 for species abbreviations. (A) Results of the BRTs for abundance with two variables week and treatment. (B) Results of the BRTs for abundance including aphid biomass as an additional variable. Here, we had to change the learning rate to 0.04 for every model regarding the plant species, because the maximum tree limit was otherwise not achieved. (C) Results of the BRTs for flowering with two variables week and treatment. (D) Results of the BRTs for flowering including aphid biomass. Models were fitted separately for each species. Cross-validation correlation (cv) is given for each model. See Supplementary Figure 6 and 7 for partial dependence plots.
