# Supplementary material for: Invertebrate Decline Leads to Shifts in Plant Species Abundance and Phenology
Source: Front Plant Sci. 2020 Sep 17;11:542125. doi: 10.3389/fpls.2020.542125 (PMC7527414; doi:10.3389/fpls.2020.542125)
Supplement: Supplementary file 6 [file Image_4.pdf]

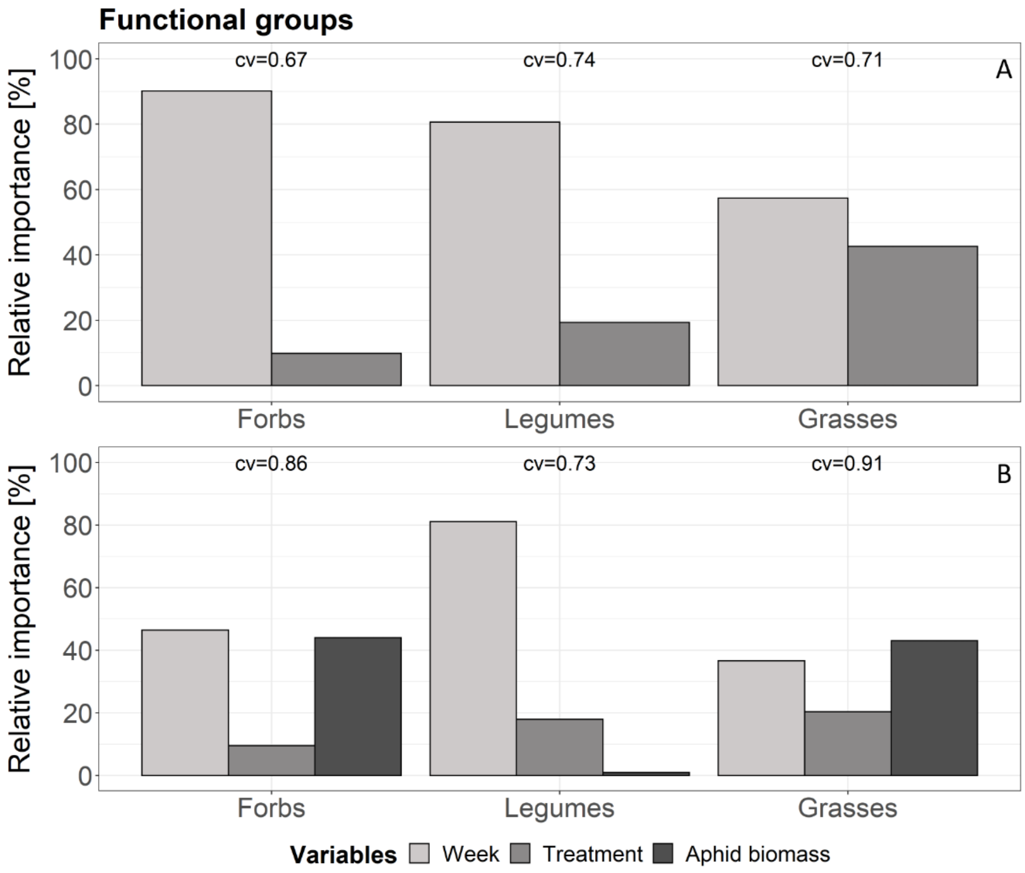

Supplementary Figure 4. Bar charts representing the relative importance of the variables week, treatment and aphid biomass for the abundance of each functional group, deduced from boosted regression tree (BRT) models, according to their abundance (432 observations). See text for parameter settings. (A) Relative importance for two variables week and treatment. (B) Relative importance including aphid biomass. Models were fitted separately for each functional group. Cross-validation correlation (cv) is given for each model. See Supplementary Figure 8 for partial dependence plots.
