# Supplementary material for: Invertebrate Decline Leads to Shifts in Plant Species Abundance and Phenology
Source: Front Plant Sci. 2020 Sep 17;11:542125. doi: 10.3389/fpls.2020.542125 (PMC7527414; doi:10.3389/fpls.2020.542125)
Supplement: Supplementary file 7 [file Image_5.pdf]

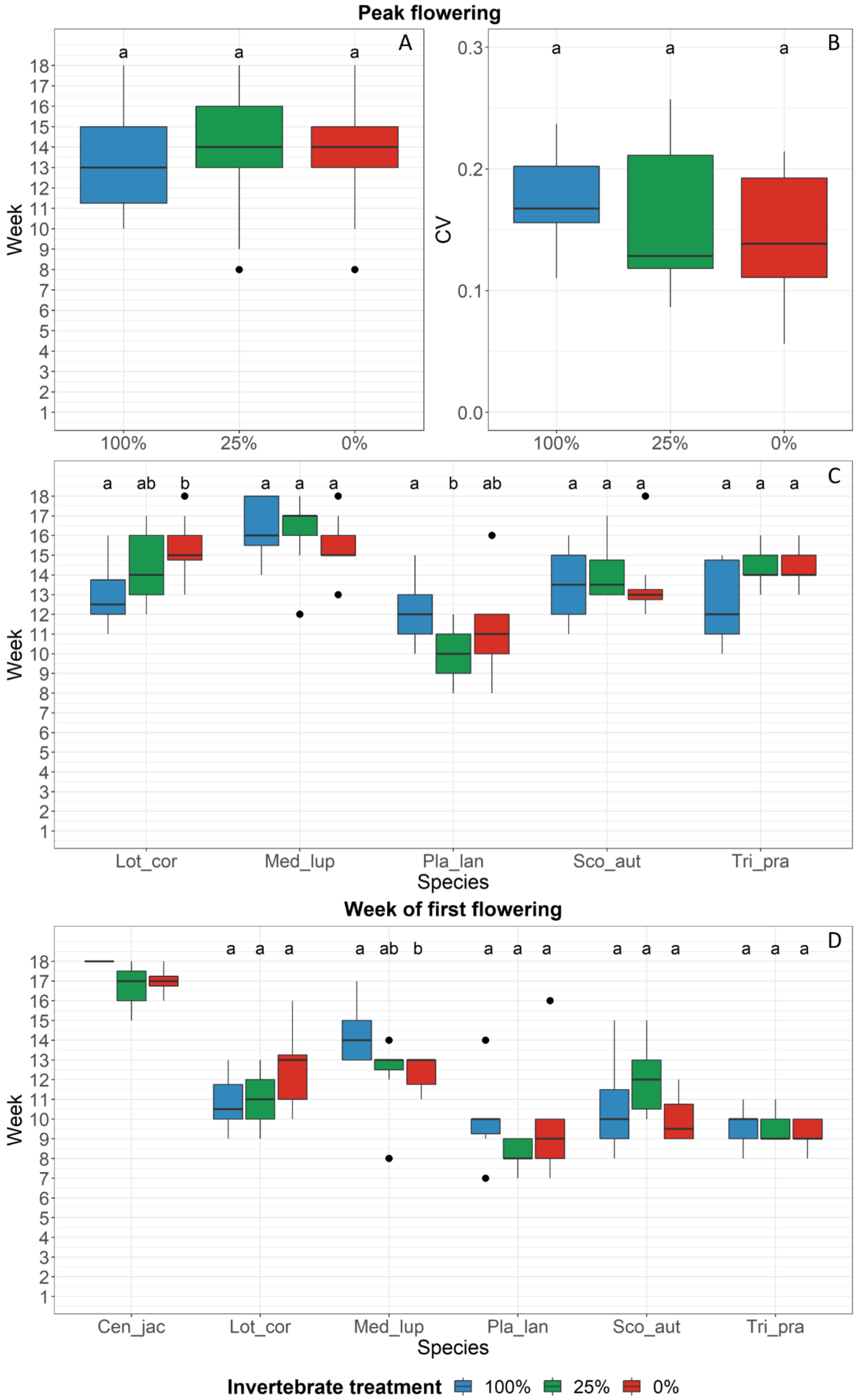

Peak flowering
A
B
C
Week
CV
100%
25%
0%
a
ab
b
Lot_cor
Med_lup
Pla_lan
Sco_aut
Tri_pra
Species
Week of first flowering
D
Cen_jac
Invertebrate treatment

Supplementary Figure 5. Boxplots showing peak flowering dispersion and week of first flowering per species and treatment; black dots mark outliers (A) Peak flowering dispersion per treatment, Kruskal-Wallis test χ^2=2.26, df=2, p=0.32; (B) Coefficient of variation per treatment, Kruskal-Wallis test χ^2=1.28, df=2, p=0.53; (C) Peak flowering for all species that reached peak flowering stage (five out of seven), Lot_cor: ANOVA F=4.33, df=2, p<0.05; Med_lup: Kruskal-Wallis test χ^2=1.93, df=2, p=0.38, Pla_lan: ANOVA F=3.21, df=2, p=0.06, Sco_aut: Kruskal-Wallis test χ^2=1.54, df=2, p=0.46, Tri_pra: Kruskal-Wallis test χ^2=4.79, df=2, p=0.09; (D) Week of first flowering per species (six out of seven), Cen_jac: no test, as only one observation for 100%-treatment, Lot_cor: ANOVA F=2.67, df=2, p<0.10; Med_lup: Kruskal-Wallis test χ^2=6.47, df=2, p=0.04, Pla_lan: Kruskal-Wallis test χ^2=2.90, df=2, p=0.23, Tri_pra: Kruskal-Wallis test χ^2=0.96, df=2, p=0.62. See Table 1 for species abbreviations.
