# Supplementary material for: Invertebrate Decline Leads to Shifts in Plant Species Abundance and Phenology
Source: Front Plant Sci. 2020 Sep 17;11:542125. doi: 10.3389/fpls.2020.542125 (PMC7527414; doi:10.3389/fpls.2020.542125)
Supplement: Supplementary file 9 [file Image_7.pdf]

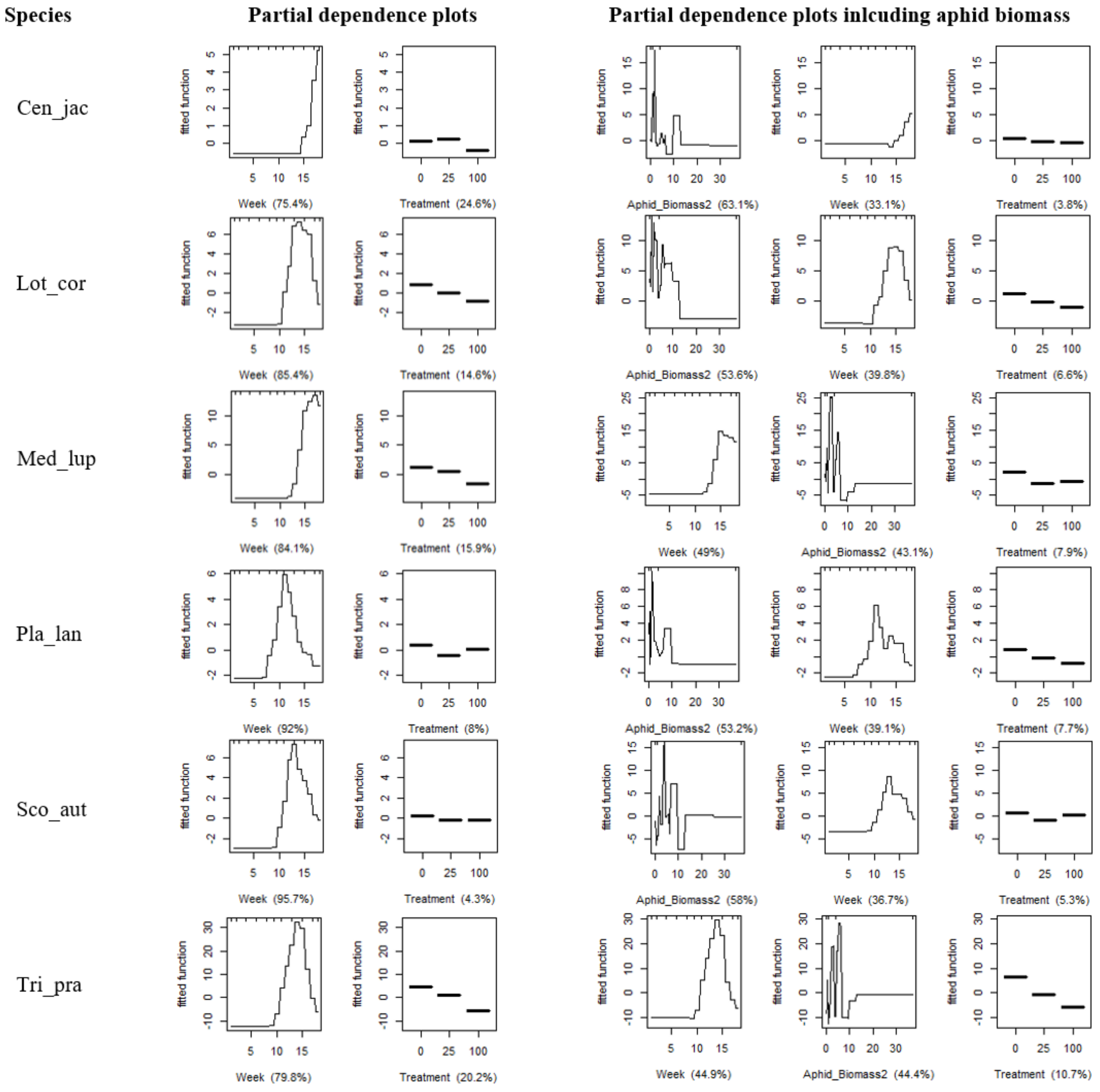

Supplementary Figure 7. Partial dependence plots from the BRT models according to the flowering phenology given for every species (see Table 1 for species abbreviations). Left plots derived from BRTs with variables week and treatment. Right plots derived from BRTs with variables week, treatment and aphid biomass.
