# Supplementary material for: Invertebrate Decline Leads to Shifts in Plant Species Abundance and Phenology
Source: Front Plant Sci. 2020 Sep 17;11:542125. doi: 10.3389/fpls.2020.542125 (PMC7527414; doi:10.3389/fpls.2020.542125)
Supplement: Supplementary file 10 [file Image_8.pdf]

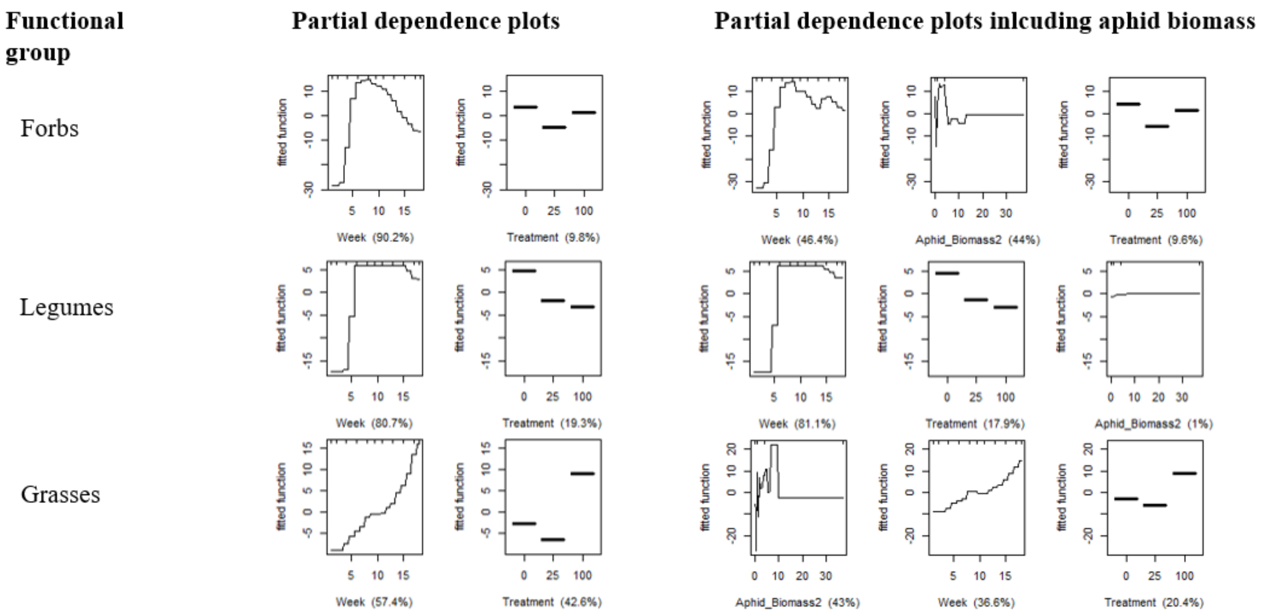

Supplementary Figure 8. Partial dependence plots from the BRT models according to the abundance given for every plant functional group. Left plots derived from BRTs with variables week and treatment. Right plots derived from BRTs with variables week, treatment and aphid biomass.
