# Supplementary material for: Invertebrate Decline Leads to Shifts in Plant Species Abundance and Phenology
Source: Front Plant Sci. 2020 Sep 17;11:542125. doi: 10.3389/fpls.2020.542125 (PMC7527414; doi:10.3389/fpls.2020.542125)
Supplement: Supplementary file 11 [file Table_1.docx]

Supplementary Table 1. Overview of plant species that were sown in the experiment with corresponding family, life form, pollination syndrome, flowering time and the information if the species flowered during the experiment. Information based on (Klotz, S., Kühn, I. & Durka, 2002) and our personal observations.

| **Species** | **Family** | **Life form** | **Pollination syndrome** | **Flower-ing time** | **Flowered in experiment** |
| --- | --- | --- | --- | --- | --- |
| *Centaurea jacea* L. s. l. | Asteraceae | Hemicryptophyte | Insects | Jun-Nov | Yes |
| *Lotus corniculatus* L. | Fabaceae | Hemicryptophyte | Insects | Jun-Aug | Yes |
| *Medicago lupulina* L. | Fabaceae | Hemicryptophyte | Insects, self-pollination | May-Oct | Yes |
| *Plantago lanceolata* L. | Plantaginaceae | Hemicryptophyte | Wind, insects, self-pollination | May-Oct | Yes |
| *Scorzoneroides autumnalis* (L.) Moench | Asteraceae | Hemicryptophyte | Insects | Jul-Sep | Yes |
| *Trifolium pratense* L. | Fabaceae | Hemicryptophyte | Insects | Jun-Sep | Yes |
| *Achillea millefolium* L. | Asteraceae | Hemicryptophyte | Insects | Jun-Oct | No |
| *Knautia arvensis* (L.) Coult. | Caprifoliaceae | Hemicryptophyte | Insects, self-pollination | Jul-Aug | No |
| *Bellis perennis* L. | Asteraceae | Hemicryptophyte | Insects, self-pollination | Jul-Nov | No |
| *Arrhenatherum elatius* (L.), P.Beauv. ex J.Presl & C.Presl | Poaceae | Hemicryptophyte | Wind, self-pollination | Jun-Oct | Yes |
| *Phleum pratense* L. | Poaceae | Hemicryptophyte | Wind, insects, self-pollination | Jun-Aug | Yes |
| *Dactylis glomerata* L. | Poaceae | Hemicryptophyte | Wind, self-pollination | May-Jul | Yes |
