# Supplementary material for: Invertebrate Decline Leads to Shifts in Plant Species Abundance and Phenology
Source: Front Plant Sci. 2020 Sep 17;11:542125. doi: 10.3389/fpls.2020.542125 (PMC7527414; doi:10.3389/fpls.2020.542125)
Supplement: Supplementary file 12 [file Table_2.docx]

Supplementary Table 2. Overview of plant-available nutrients that were examined in the soil solution. The means and standard deviations per invertebrate treatments are in mg/l. The L-ratio and the p-value give the results of the LME testing for the significance of invertebrate treatment, the four time steps when soil solution was sampled and the interaction between invertebrate treatment and time.

|  | **Invertebrate treatment [mean (+ SD)]** | | | **LME results [L-ratio (P-value)]** | | |
| --- | --- | --- | --- | --- | --- | --- |
|  | **0%** | **25%** | **100%** | **Invertebrate treatment** | **time** | **Invertebrate treatment × time** |
| NO_3_^-^ | 2.55 (± 5.45) | 3.80 (± 4.95) | 4.13 (± 4.94) | 0.5 (0.779) | **17.9 (< 0.000)** | 0.7 (0.714) |
| NH_4_^+^ | 0.02 (± 0.01) | 0.02 (± 0.00) | 0.02 (± 0.00) | 1.7 (0.418) | 0.6 (0.440) | **6.0 (0.049)** |
| PO_4_^3-^ | 0.09 (± 0.09) | 0.08 (± 0.08) | 0.07 (± 0.07) | 0.4 (0.808) | 1.4 (0.242) | 2.6 (0.275) |
| K^+^ | 2.91 (± 1.14) | 6.81 (± 11.03) | 4.96 (± 3.05) | 1.5 (0.464) | 3.1 (0.079) | 0.5 (0.762) |
